# Investigations on the effect of different influencing factors on film cooling effectiveness under the injection of synthetic coolant

**DOI:** 10.1038/s41598-021-83080-9

**Published:** 2021-02-09

**Authors:** Jianlong Chang, Xinlei Duan, Yang Du, Baoquan Guo, Yutian Pan

**Affiliations:** 1 College of Mechatronic Engineering, North University of China, Taiyuan, 030051 China; 2 Institute of Military-Civilian Integration and Innovation, North University of China, Taiyuan, 030051 China

**Keywords:** Aerospace engineering, Mechanical engineering

## Abstract

By combining the synthetic jet and film cooling, the incident cooling flow is specially treated to find a better film cooling method. Numerical simulations of the synthetic coolant ejected are carried out for analyzing the cooling performance in detail, under different blowing ratios, hole patterns, Strouhal numbers, and various orders of incidence for the two rows of holes. By comparing the flow structures and the cooling effect corresponding to the synthetic coolant and the steady coolant fields, it is found that within the scope of the investigations, the best cooling effect can be obtained under the incident conditions of an elliptical hole with the aspect ratio of 0.618, the blow molding ratio of 2.5, and the Strouhal number *St* = 0.22. Due to the strong controllability of the synthetic coolant, the synthetic coolant can be controlled through adjusting the frequency of blowing and suction, so as to change the interaction between vortex structures for improving film cooling effect in turn. As a result, the synthetic coolant ejection is more advisable in certain conditions to achieve better outcomes.

## Introduction

Modern gas turbine engines typically are operated at combustion chamber with the temperatures in excess of 1500 K, some even higher^1^, which can cause damage to the engine. Therefore, engine components in the thermal path must be protected. Currently the most widely used method is to gas film cooling technology, by cooling the relatively cold gas components in the hole into the stream. Then, cooling gas on the downstream side of the hole to form a layer of low temperature "thin film", thus cooling parts surface. At present, the use of steady flow for film cooling was most applied. Therefore, in this manuscript, synthetic jet is adopted in film cooling technology, to find a better way of film cooling.

Synthetic jet is widely used in aerospace, including vector jets, separation control, enhanced mixing, and reduction of wall friction^2–5^. Synthetic jet is a way to achieve active control by governing the development and fusion of vortex coherent structures. Being applied in many fields, synthetic jet can be controlled by managing the stable blowing or suction frequency. Synthetic jet is of great practical value^6–8^, because it does not require complex fluid pipeline system. Many scholars have done a lot of researches on film cooling of unstable jets as a coolant, which mainly includes the pulsating coolant.

The results of Aga et al.^9^ showed that the pulsating jet had greater thermal mixing and changed the size of the jet. Due to the greater penetrating power near the hole and the effective blowing ratio change, the entropy loss of the jet pulsating along the flow was twice as large as the entropy loss of the stable jet. Bernsdorf^10^ studied the hypothesis of the quasi-steady state mode of pulsating coolant injection. The conclusion on the validity and limitations of this hypothesis was based on the results of the pulsating velocity field, and the amplitude of the pulsation was found to be the limiting parameter. In order to explore the effects on film efficiency and heat transfer from jet pulsation and duty cycle, the jet pulsation frequencies of 5 Hz, 10 Hz and 20 Hz with a film hole on the circular front edge of the bluff body were studied^11^. The results showed that the reduced blowing ratio can achieve higher efficiency and lower heat transfer coefficient. The film effectiveness results presented in the research^12^ showed that the effectiveness of a pulsating flow was lower at VR of 1 and 2, pulsating frequency 10 Hz and duty cycle 50% than a continuous film flow. When the blowing ratio was 0.5 and 1.5, and the Strouhal number ranged from 0.0119 to 1.0, the film cooling of the pulsating plate was simulated through the realizable k-ε turbulence model^13^. The research results showed that the performance of the pulse jet depended largely on the geometry and blowing ratio. In order to verify whether the pulsation of the coolant jet can enhance the film cooling effect, Muldoon^14^ conducted direct numerical simulation (DNS) on the pulsed film cooling jet. The results showed that at the same peak blow ratio (M = 1.5), the pulse produced an increase in the overall film cooling efficiency compared to the continuous flow. It should be noted that pulses may be an optional effective way to enhance the cooling effect of the film in many practical configurations.

Ou^15^ had done the studies on the effects of the coolant jet duty cycle (DC), and hole shape geometry on heat transfer coefficient and film effectiveness with a film hole located on a semicircular leading edge test model with an afterbody. The investigations meant that the pulsed coolant performed better than the continuous coolant, except for a small region near the film hole despite hole geometry and blowing ratio. A test device was used by Sultan^16^, which combined an injection method from a single cylindrical hole inclined at 30° to a thermally uniform mainstream, to determine the flow structure change due to the pulsation of the injection agent. It had been found that as the separation of the jet from the wall became apparent (M = 1 and 1.25), the pulsation improved the wall coverage of the ejected fluid under low frequency excitation. Sultan^17^ investigated the effects of sinusoidal pulsations externally applied to oblique circular jets. In his investigation, the velocity field measured by the time-resolved particle image velocimetry system (TR-PIV) as well as adiabatic wall temperature and convective heat transfer coefficient, were analyzed to determine the effect of spray film modulation. It could be observed that in the case of stable blowing, when the airflow was still attached to the wall, the pulsation often leads to a decrease in efficiency.

An experimental study of a pulsed film cooling jet subjected to periodic wakeups was performed by Womack^18^. The cases of a single row of cylindrical film cooling holes inclined by 35° with respect to the surface, with blow ratios of 0.50 and 1.0 and Strouhal number of 0.15, 0.30, and 0.60. The results showed that at low Strouhal numbers, there was a significant effect for the wake-up time on the instantaneous film cooling effectiveness, but usually the wake timing had little effect on the average time efficiency. The experimental study^19^ of the periodically awakened film cooling flow was carried out under conditions similar to those of Womack^18^. The results showed that the heat transfer coefficient increased with the increase of wake flow frequency.

At present, there are many investigations on the film cooling of the pulsating and steady coolant, and the film cooling of the synthetic coolant is hardly involved. The main difference between the synthetic coolant and the steady coolant is that the net mass flow of the synthetic coolant through the orifice is zero, which means that there’s no change in gas mass flux during blowing and suction process. The flow of a steady coolant includes the cooling fluid and the surrounding environmental fluids entrained by the coolant, while the synthetic coolant is composed of the environmental fluids only. For the film cooling, compared with steady coolant, the synthetic coolant with similar average velocities can achieve stronger mixing effects by providing an equivalent increase in momentum at a smaller mass flow rate. At the same time, the shear layer of the synthetic coolant is thicker, which helps delay the formation of vortices. In addition, the synthetic coolant enjoys stronger penetrability and can increase the vertical momentum of the coolant, thereby effectively reducing the crossflow fluid's entrainment to the near field. When the crossflow is driven by the synthetic coolant, structural changes occur. And since the synthetic coolant is controllable, the effect of the structures to impede the formation of the film in the steady coolant flow field may be markedly attenuated in the synthetic coolant flow field. Considering that the velocity distribution of the synthetic coolant is a function of time and space, the sine function is employed here to set the velocity of the cooling coolant at the orifice in the boundary condition, as followed in Eq. (1).

1 $$V(t,y) = U_{\max } g(y)\sin (2\pi ft),u(t,x) = 0$$

where $U_{\max }$ is the maximum velocity, and $g(y)$ is the distribution function of the coolant in the streamwise direction. In this manuscript, $g(y) = 1$.

The calculation formula of cooling efficiency is:

2 $$\eta = \frac{{T_{m} - T_{P} }}{{T_{m} - T_{C} }}$$

Among them, *T*_*m*_ is the mainstream temperature, *T*_*p*_ is the wall temperature, and *T*_*c*_ is the temperature of the coolant. The calculation formula can also be understood as a measure of the degree of cooling flow spreading and protecting the wall.

## Physical model and numerical methods

### Physical model and meshes

The dimensions of the calculation domain in the axial direction, wall normal direction and span direction (*x*, *y*, and *z*) are 50D × 20D × 20D. When setting initial boundary conditions, the crossflow is uniformly passing through the calculation domain, and the coolant with a velocity function with Eq. (1) is conducted. The coolant is changed from a steady coolant to a synthetic coolant. The diameter D of the air film hole is set to a unit length, and the different blowing ratio is taken by calculating the ratio of the average velocity of the coolant to the mainstream velocity. In order to offer enough space for the coolant to fully develop, the length of the cooling pipe is 6D. What’s more, in order to reduce the influence of the outlet environment near the gas film hole on the fluid, the distance between the orifice and the lateral mainstream outlet in the *x* direction is 5.5D.

The velocity of the boundary layer at the inlet of the crossflow is uniformly distributed. For a constant crossflow with Reynolds number of 100,000, the inlet velocity of the crossflow can be reversed according to the calculation formula of the Reynolds number, and the inlet velocity of the high-temperature crossflow can be deduced. The calculation formula of Reynolds number is:

3 $$Re = \frac{\rho vd}{\mu }$$

Among them, ${\uprho }$ refers to the fluid density, v refers to the characteristic velocity of the flow field, d is the characteristic length of the flow field, and ${\upmu }$ is the dynamic viscosity coefficient of the fluid. In this paper, the fluid density is 1.225 kg/m^3^, the characteristic length of the flow field is 28D, and the dynamic viscosity coefficient of the fluid is 1.7894e−05 N s/m^2^.

**Figure 1:**
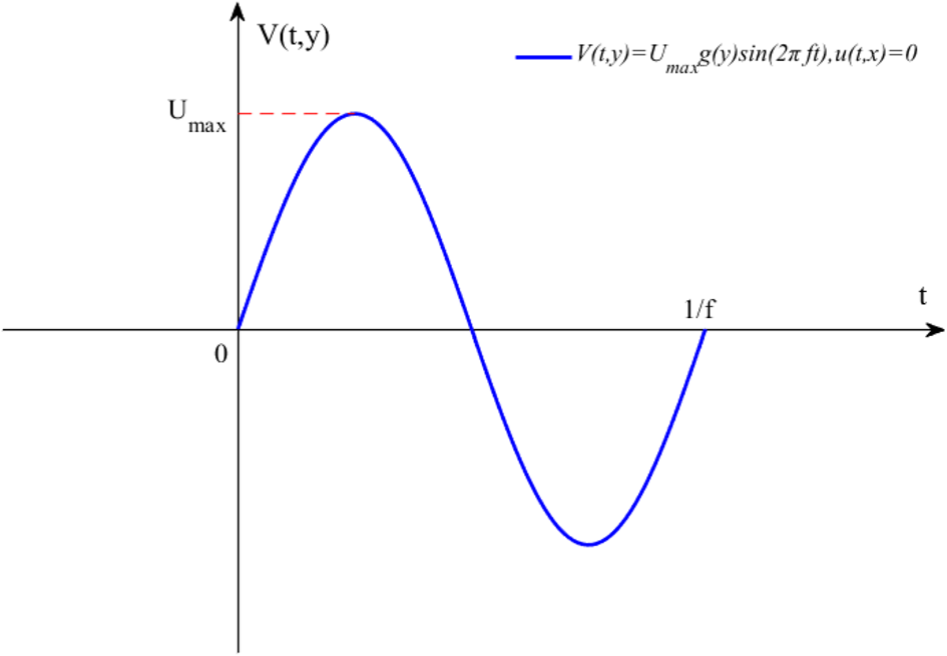
Velocity profile for Eq. (1).

The temperature of the high temperature crossflow is 1500 K, and the temperature of the coolant is the room temperature of 293 K. Based on the calculation formula of the blowing ratio, the average velocity of the coolant can be acquired, and the instantaneous velocity of the coolant at each moment can also be obtained by combining Eq. (1). Velocity profile for Eq. (1) is shown in Fig. 1.

The physical model adopted in the simulation is a 3D transient model, in which the crossflow evenly flows through the computational domain. All the wall surfaces are set to be adiabatic. The incidence angles relative to the crossflow direction are specified to 30°, 60°, 90°, respectively. The length of the entire computational domain is 50D. Consequently, the length of the mixing area of the crossflow and the cooling flow is 44.5D, the width and height of the computational domain are both 20D.

**Figure 2:**
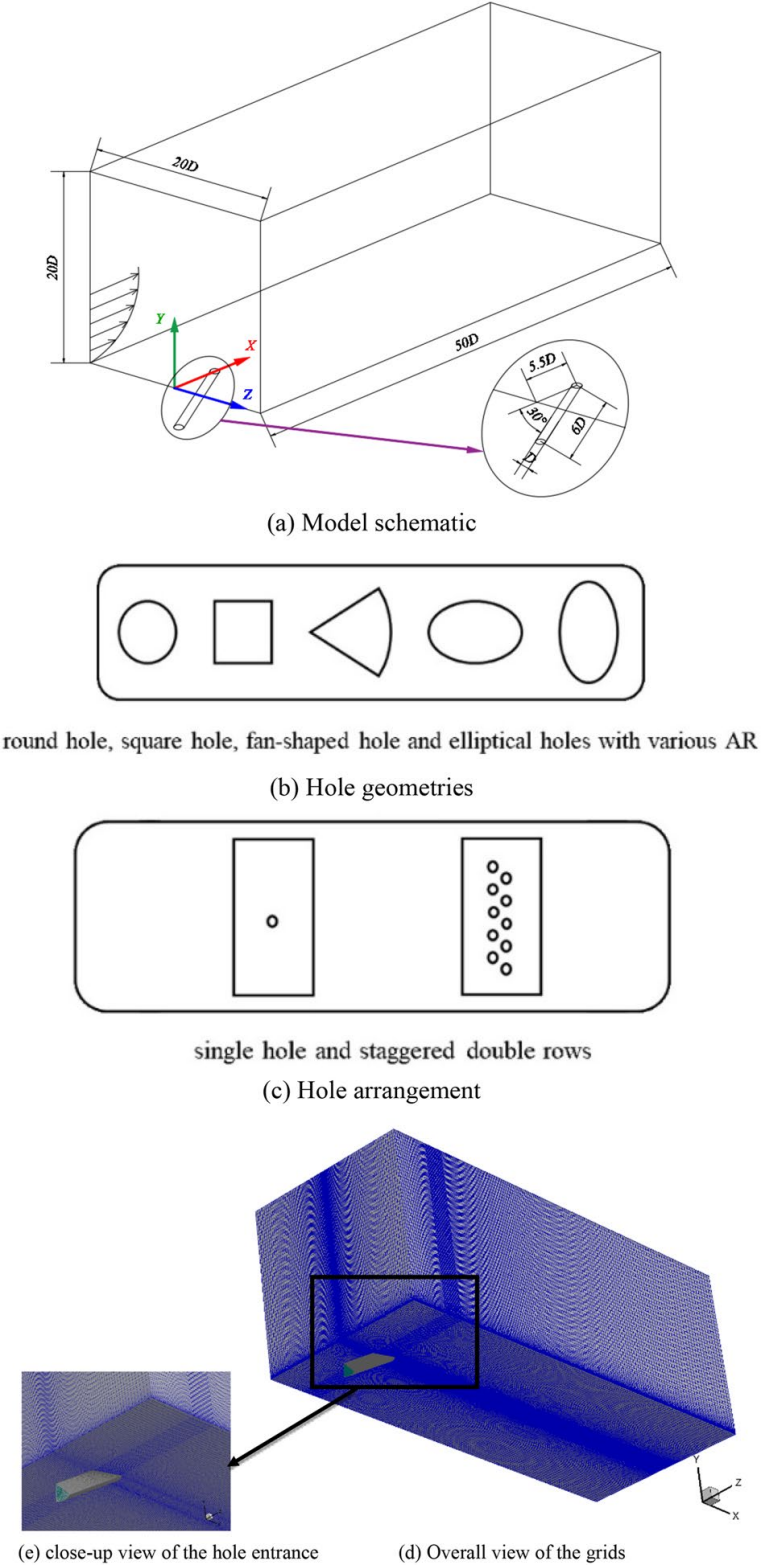
Physical models of different situations and structures of the grids.

The physical models corresponding to different hole geometries, and different hole arrangements are shown in Fig. 2.

The influence of thermal and geometric parameters such as the angle of incidence, hole pattern, hole arrangement, blowing ratio, and Reynolds number on film cooling flow field need to be further scrutinized. A wide range of these specific parameters are selected for the numerical simulation of film cooling. The flowing direction of crossflow on the horizontal *x* direction is plotted against the other component of the coolant on the vertical *y* axis, and the spanwise direction of the film is on the *z* axis.

In this manuscript, ICEM is used for the structured grids generated. Since the subsequent finite element analysis software Fluent adopts the finite volume method which requires completely unstructured grids, the generated structured grids are supposed to convert into unstructured grids. The computational domain established in this manuscript consists of approximately 900,000 hexahedral elements as displayed in Fig. 2d. The close-up view of the hole entrance was shown in Fig. 2e. For the sake of better decomposing the fluid and boundary layer, the grid cells near the wall and the orifice should be set small enough to ensure a seamless connection with the wall. At the same time, the grids away from the wall are ought to be smoothly transitioned to the larger ones to reduce the amount of calculation. The structure of the grids in the whole flow field is generated as follows: the size of the cell around the orifice increases linearly outward at a ratio of 1.05 until the size of the cell grows to the aperture D.

In order to capture the viscous sublayer, mesh refinement is performed near the wall to ensure the *y*^+^ value near the wall being less than or close to the unit length. And with the purpose of improving the calculation accuracy, the ratio of two adjacent grids at any position is kept in the range of 0.7–1.3. This undertaking is used to obtain the optimal grid size and get the solution of grid independence in turn. The large eddy simulation method is applied for the grid-independent test. As a prerequisite, it is assumed that through utilizing this simulation method, the grids generated for all other turbulence models would get grid-independent solutions.

### Numerical methods

Konopka^20^ showed that the LES was suitable for solving three-dimensional unsteady compressible Navier–Stokes equations in film cooling. That means that LES is suitable for the interaction between fluids in the current film cooling problem. At the same time, Puggelli^21^ stated that LES could clearly show the effect of countercurrent on film cooling effectiveness during film cooling. Therefore, LES is more appropriate for predicting flow fields with large coherent structures as the main feature. In the crossflow field, it is very important to predict the unsteady behavior of various flow structures. Compared with RANS, the non-steady-state LES with space-time resolution can provide a more suitable model.

The aim of the LES is to resolve the larger scale of turbulence, and the smaller ones are modeling based on the universality. Therefore, the first step to realize LES is to filter out small-scale pulsations and the filtered NS equation is obtained as the following equation:

4 $$\left\{ {\begin{array}{*{20}l} {\frac{{\partial \overline{{u_{i} }} }}{\partial t} + \frac{{\partial \overline{{u_{{\text{i}}} u_{{\text{j}}} }} }}{{\partial x_{j} }} = \frac{1}{\rho }\frac{{\partial \overline{p} }}{{\partial x_{i} }} + \nu \frac{{\partial^{2} \overline{{u_{i} }} }}{{\partial x_{i} \partial x_{j} }}} \hfill \\ {\frac{{\partial \overline{{u_{i} }} }}{{\partial x_{i} }} = 0} \hfill \\ \end{array} } \right.$$

where $\overline{{u_{{\text{i}}} u_{{\text{j}}} }} = \overline{{u_{{\text{i}}} }} \overline{{u_{{\text{j}}} }} + (\overline{{u_{{\text{i}}} u_{{\text{j}}} }} - u_{{\text{i}}} u_{{\text{j}}} )$, $- (\overline{{u_{{\text{i}}} u_{{\text{j}}} }} - u_{{\text{i}}} u_{{\text{j}}} )$ is the sub-lattice stress, then the NS equation is:

5 $$\frac{{\partial \overline{u}_{i} }}{\partial t} + \frac{{\partial \overline{u}_{i} \overline{u}_{j} }}{{\partial x_{j} }} = - \frac{1}{\rho }\frac{{\partial \overline{p} }}{{\partial x_{i} }} + \nu \frac{{\partial^{2} u_{i} }}{{\partial x_{i} \partial x_{j} }} - \frac{{\partial (\overline{{u_{{\text{i}}} u_{{\text{j}}} }} - \overline{{u_{{\text{i}}} }} \overline{{u_{{\text{j}}} }} )}}{{\partial x_{j} }}$$

The right sub-lattice stress term is not closed. To realize LES, a closed pattern of sub-lattice stress must be constructed. Common sub-lattice stress modes include Smagorinsky eddy viscosity mode, scale similarity mode, mixed mode, dynamic mode, spectral space eddy viscosity mode, structure function mode and CZZS mode.

The sub-lattice stress mode selected in this manuscript is the Smagorinsky eddy viscosity mode. The eddy viscosity sub-lattice model of this model is dissipative. In the case of isotropic filtering, it meets the constraints of the model equation. In addition, the Smagorinsky model performs good adaptability for the calculation program for viscous fluid motion. It is the first sub-lattice stress model to be applied to LES in the atmosphere and engineering. Assuming that the isotropically filtered small-scale pulsation is locally balanced, the Smagorinsky eddy viscosity mode is equivalent to a mixed-length form of vortex-viscosity mode, that is

6 $$\overline{\tau }_{ij} = (\overline{{u_{i} }} u_{j} - \overline{{u_{i} u_{j} }} ) = 2(Cs\Delta )^{2} \overline{S}_{ij} (2\overline{S}_{ij} \overline{S}_{ij} )^{1/2} - \frac{1}{3}\overline{\tau }_{kk} \delta_{ij}$$

where Δ is the filtering scale, $v_{t} = (C_{s} \Delta )^{2} (\overline{S}_{ij} \overline{S}_{ij} )^{1/2}$ is subgrid eddy viscosity coefficient, and $C_{s}$ is Smagorinsky constant.

The LES equation for filtered scalar transport is:

7 $$\frac{{\partial \overline{\theta } }}{\partial t} + u_{i} \frac{{\partial \overline{\theta } }}{{\partial x_{i} }} = k\frac{{\partial^{2} \overline{\theta } }}{{\partial x_{k} \partial x_{k} }}\underbrace {{ + \frac{\partial }{{\partial x_{i} }}(\overline{{u_{i} }} \ddot{\theta } - u_{i}^{^{\prime}} \theta ^{\prime})}}_{{{\text{Sub - lattice}}\,{\text{scalar}}\,{\text{transport}}}}$$

Among them, the sub-lattice scalar transport is a quantity that needs to be closed for LES. After filtering small-scale pulsations, the governing equations for large-scale motion are derived.

### Model verification of accuracy

**Figure 3:**
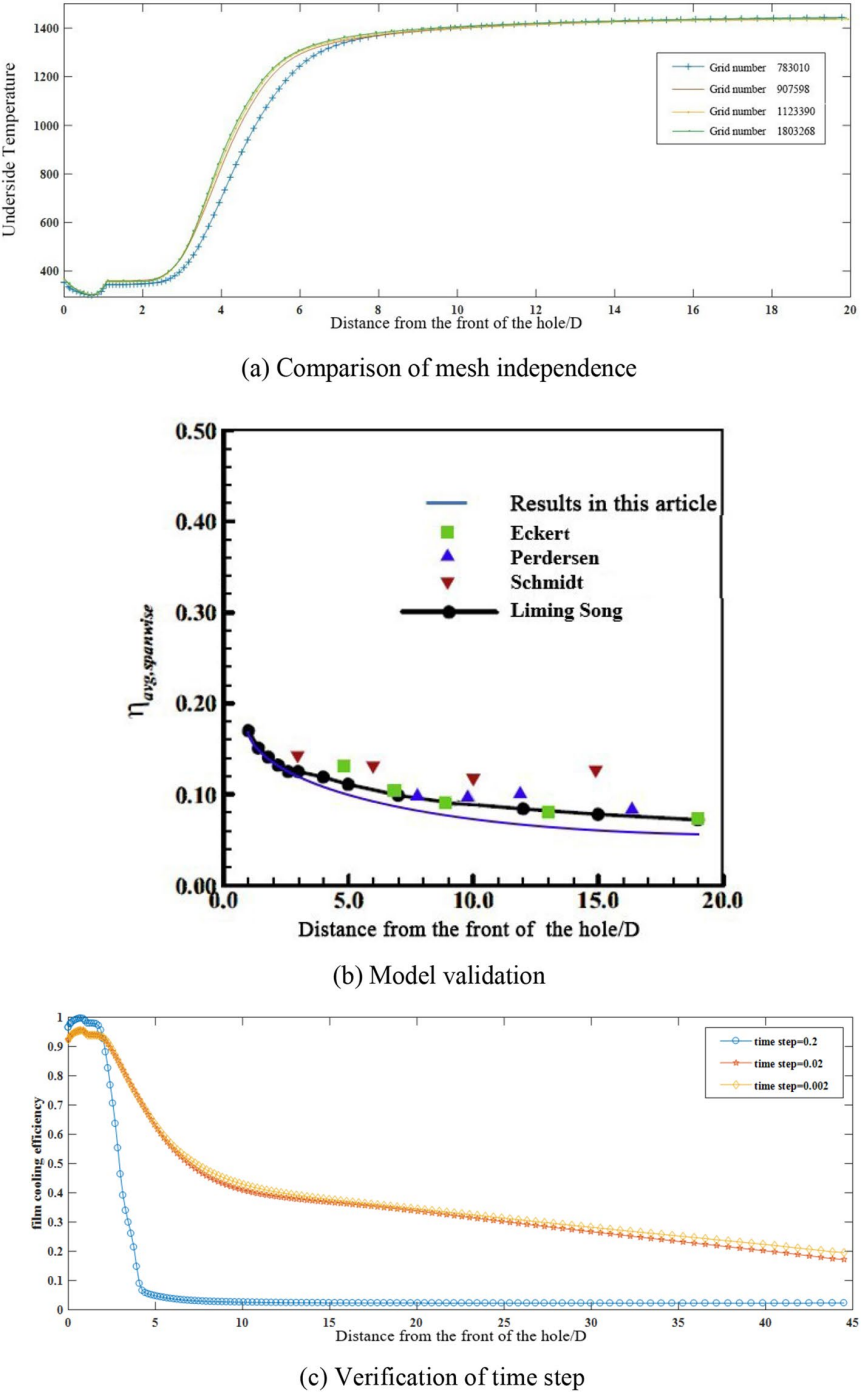
Model verification of accuracy.

The grid-independence test is conducted with the 30° incidence from a single round hole as the reference setting parameter. In the calculation domain of 50D × 20D × 20D, the grid numbers are respectively taken as 783010, 907598, 1123390 and 1803268. Different bottom surface temperature curves are acquired from simulation results of the four grid numbers. Meanwhile, the ratio of the distance from the orifice along the flow direction to the aperture is taken as abscissa, as illustrated in Fig. 3.

Figure 3a shows the results of grid independence validation. The flow field region of 0 < *x*/*D* < 20 is intercepted for analysis. For the single round hole, 30° incidence, there is relatively large deviation between the grid number of 783010 and the number of 907598. While smaller deviation occurs between the grid number of 907598, 1123390 and 1803268. From the above results, it can be concluded that when evaluating the film cooling effect, the grid number of 907,598 is sufficient for the flow field analysis. Further increasing the grid number does not significantly change the results, but waste computing resources. Therefore, the grid number of 907598 is selected for numerical simulation, where *y*^+^ near the wall does not exceed 1. For the grid numbers of the other cases investigated in the manuscript, they should be close to 907598. Besides, the grid numbers of double staggered-row incidence are respectively 1529642.

In the cause of verifying the existing calculation model, the working conditions are set as the blowing ratio M = 1, and single round hole, 30° incidence. Eckert^22^, Pederson^23^, and Schmidt^24^ had performed experiments under the same conditions, and corresponding results are attained. Moreover, based on detailed experimental investigation on flow mechanism and thermal characteristics of film cooling, Song^25^ researched the impact of blowing ratio and inclination angle on the film cooling effectiveness enhancement by using vortex generator. Numerical simulations are performed by LES in this manuscript, and the results are displayed in Fig. 3b. Our simulation parameters are compared with the experimental results mentioned.

From Fig. 3b, it can be revealed that the simulation results by LES fit well with the experimental results. This simulation reproduces the trend of the film cooling effectiveness curve along downstream. Although the numerical simulation values at the position 0 < *x*/*D* < 10 are slightly larger than the experimental results, the maximum error is still within the acceptable range. We believe that there are two reasons for this difference. First, the experimental injecting conditions are less ideal than the simulating conditions, the differences in boundary layer thickness, jet velocity profile, geometrical variations, and turbulence may lead to the change of the results. Second, LES may have underestimated the penetrability of the jet. According to the fitting between the numerical simulation results and experimental results, it can be deduced that the model adopted in the manuscript is reasonable.

According to the calculation formula of the time step, the approximate value of the time step should be the ratio of the minimum grid size to the velocity of the fluid. The time step is employed as 0.2 s here. Hence, the time steps are taken respectively as 0.2 s, 0.02 s, and 0.002 s, the comparison of the film cooling effectiveness curves is inferred in Fig. 3c. According to the comparison between curves in Fig. 3c, it can be discovered that numerical simulation results of the time step 0.2 s are very different from the other two, indicating that the complexity of the flow field and flow structures is seriously underestimated. Therefore, the time step is further reduced to 0.02 s and 0.002 s, ant it can be arrived at that the difference between the calculation results of the latter two is very small and almost negligible. In view of the fact that more computing resources would be consumed under the time step being 0.002 s, as a result, the time step is set as 0.02 s.

By performing the verifications of grid independence, model verification, and time step, the final numerical simulation method is identified as the large eddy simulation (LES). The verification of the model proved that the selection of the calculation model is reasonable; the number of grids is 907598, under which reliable data can be obtained without wasting computing resources. The picked time step is 0.02 s, for the flow field structures calculated by this time step are accurate enough.

## Results and discussion

In this section, based on the influence of different factors on the flow field and the film cooling effectiveness when the steady coolant is ejected, the film cooling results when the synthetic coolant is ejected under different hole shapes, different blowing ratios and different Strouhal numbers are explored.

### Film cooling effectiveness of synthetic coolants under different blowing ratios

**Figure 4:**
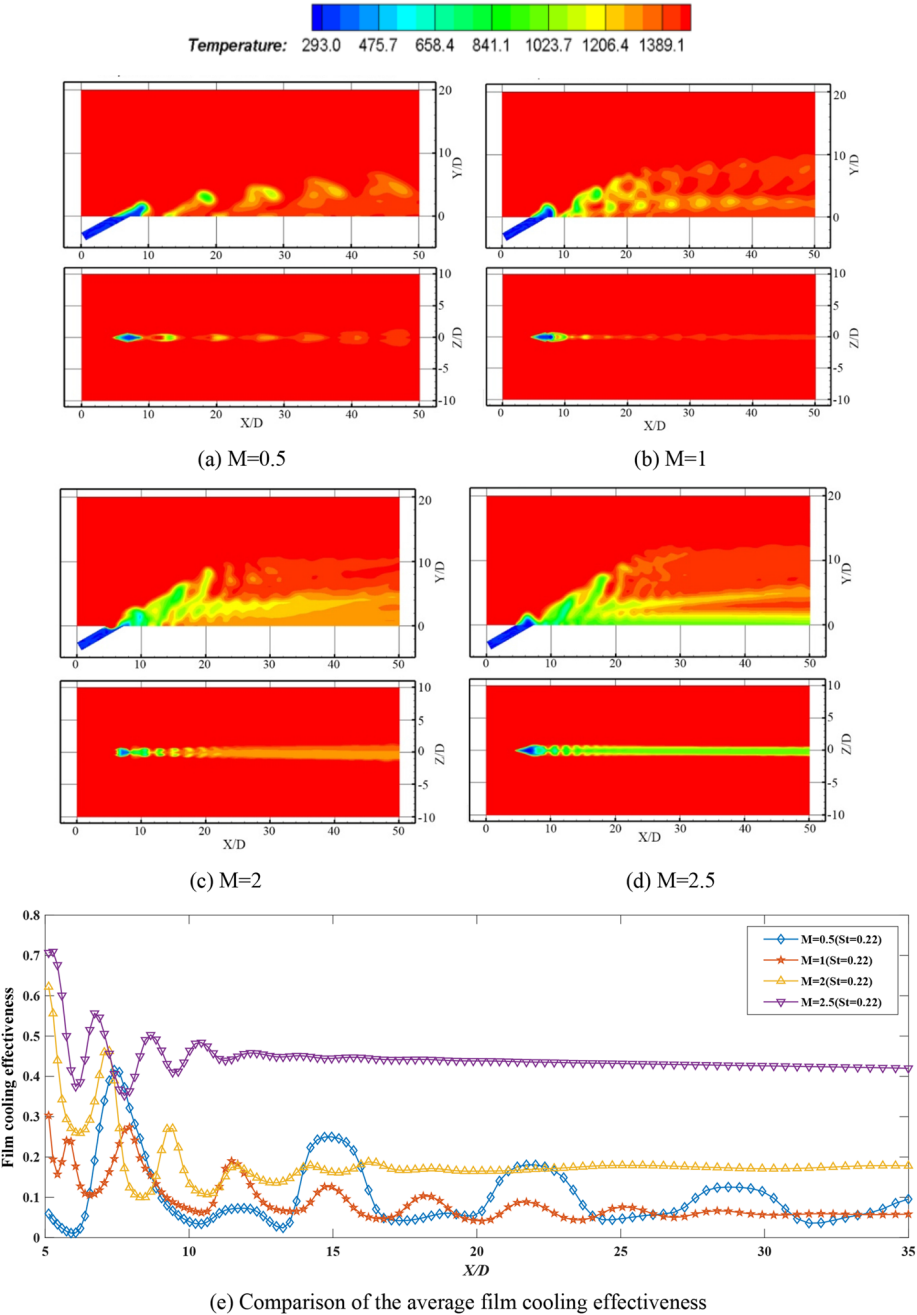
Temperature contours and comparison of the average film cooling effectiveness of synthetic coolants at different blowing ratios at *St* = 0.22 and fan-shaped hole.

Among the cases investigated in the manuscript, when the effect of different blowing ratios is discussed, the fan-shaped hole is selected as the incident hole shape, and the Strouhal number is fixed at 0.22, and the blowing ratios are respectively taken as 0.5, 1, 2 and 2.5. The simulation results obtained are illustrated in Fig. 4.

**Figure 5:**
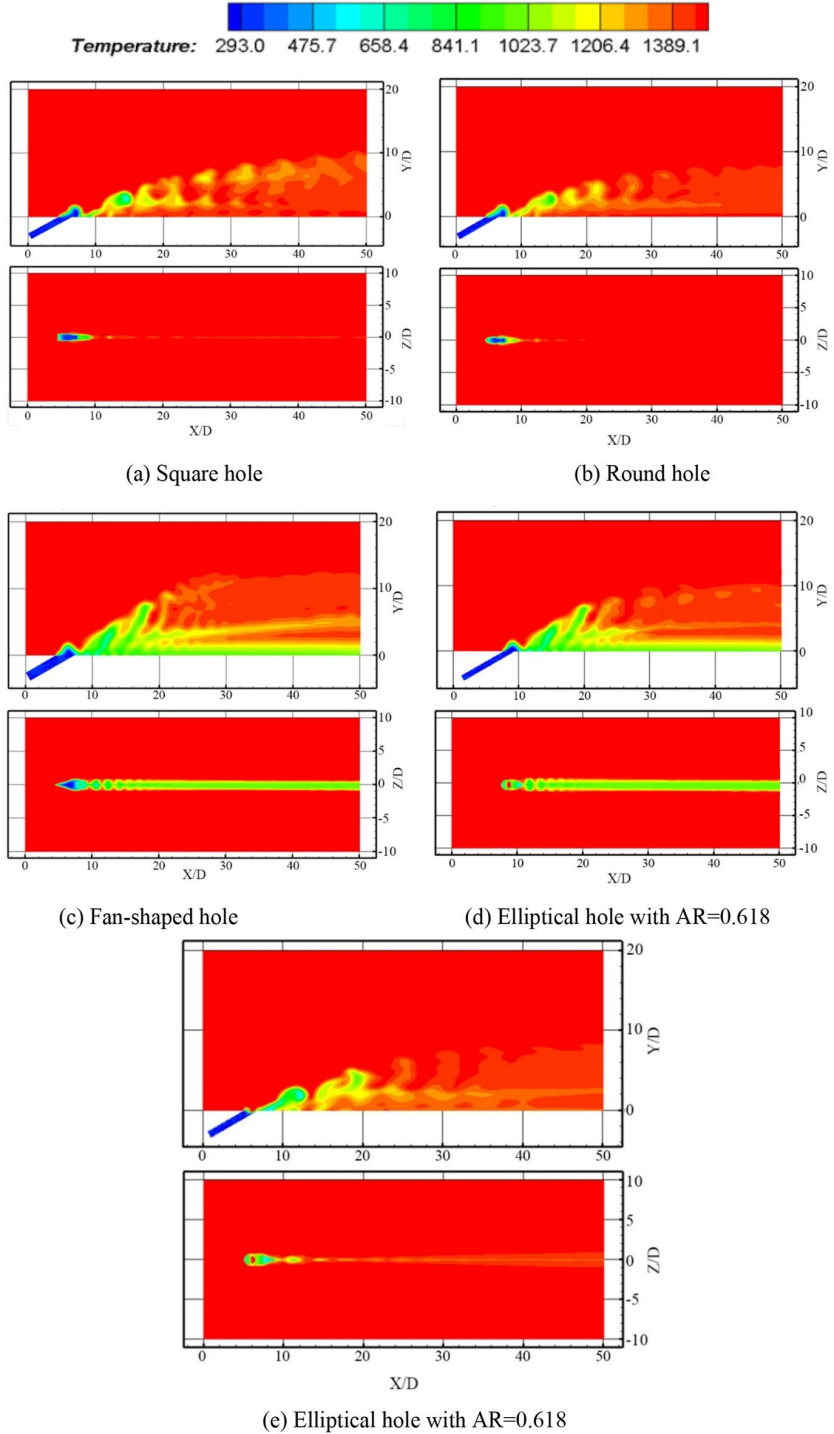

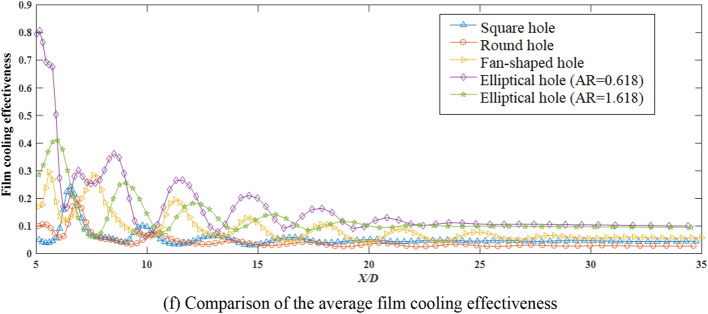
Temperature contours and comparison of the average film cooling effectiveness of synthetic coolants with different apertures incident.

**Figure 6:**
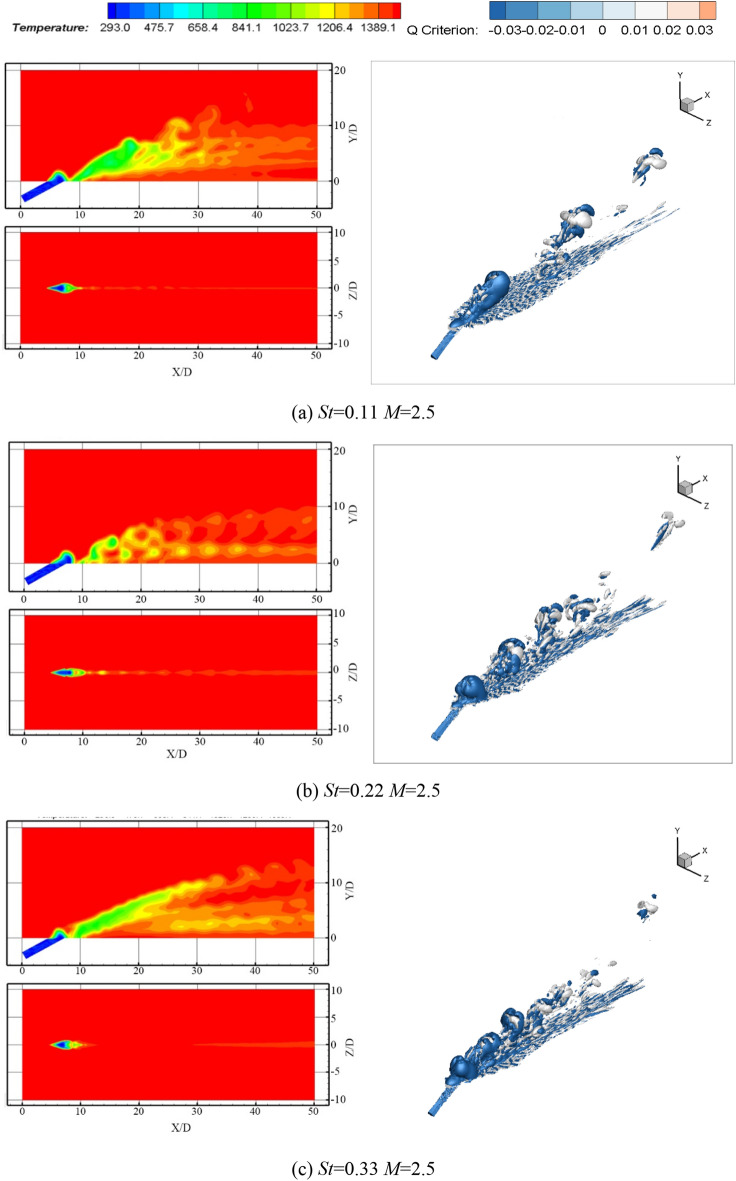

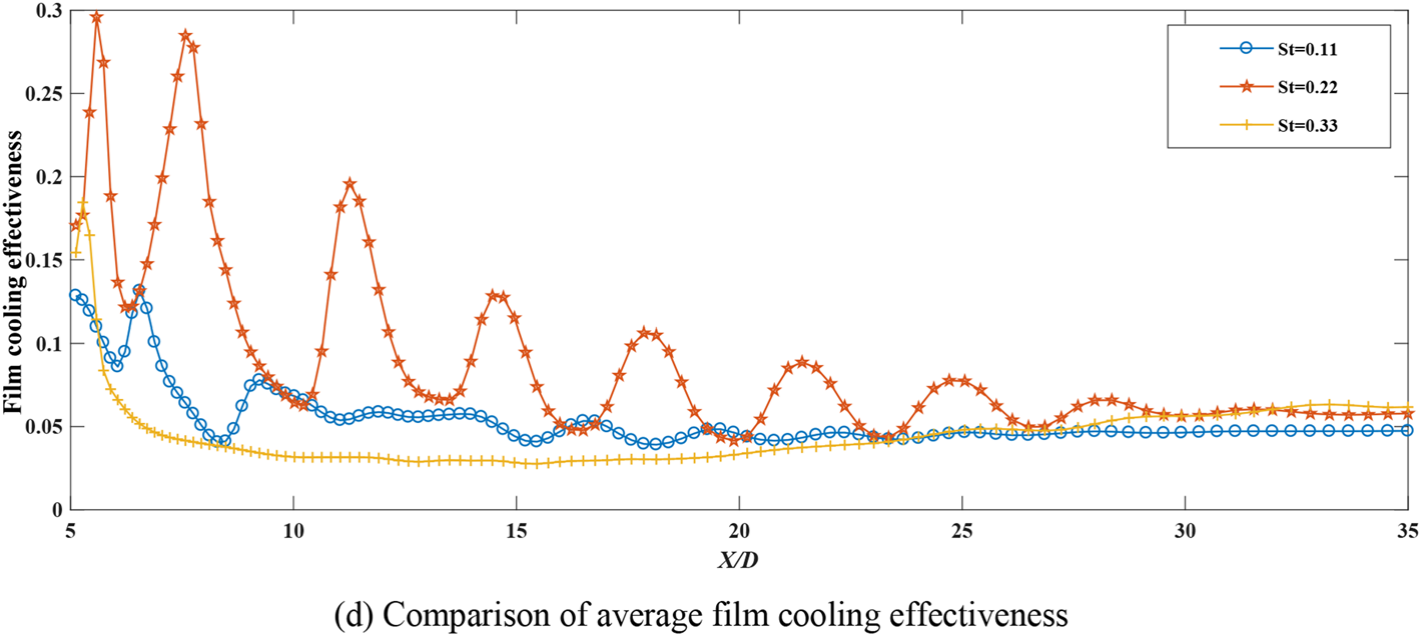
Temperature contours and comparison of average film cooling effectiveness when a synthetic coolant is ejected at different Strouhal numbers with fan-shaped hole and *M* = 2.5

**Figure 7:**
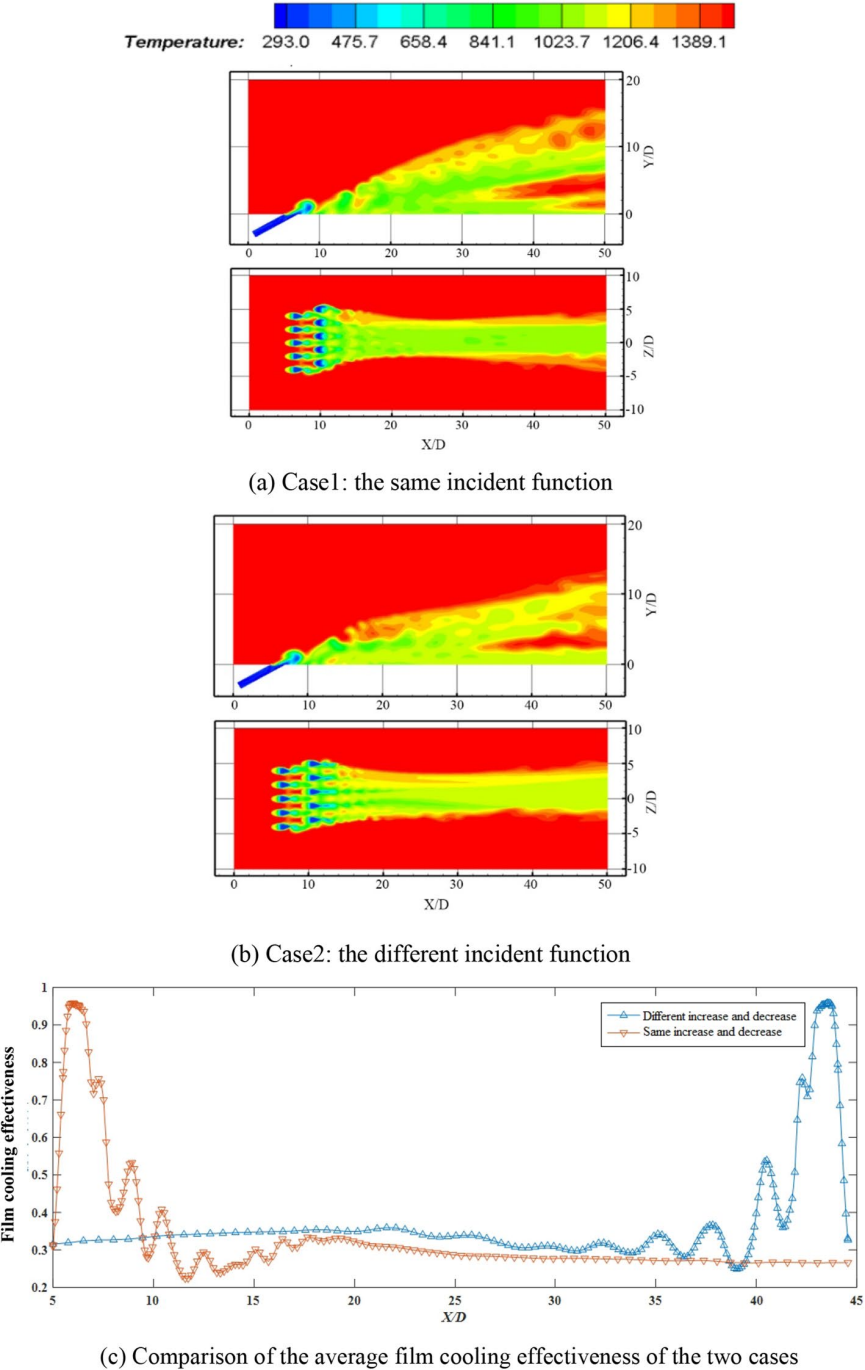
Temperature contours and comparison of the average film cooling effectiveness of the two incident modes of incident modes of the inserted dual-row at 30° incidence and *St* = 0.22.

Regarding the two temperature cloud diagrams in the manuscript (include Figs. 4, 5, 6, 7), the upper one is the middle section through the hole, that is, the *Z* = 0.001D section, and the other is the film cooling wall, that is, the *Y* = 0.001D section.

**Table 1:** The insertion function at two cases.

	Left hole incident function	Right hole incident function
Case1	$V(t,y) = U_{\max } g(y)\sin (2\pi ft),u(t,x) = 0$	$V(t,y) = U_{\max } g(y)\sin (2\pi ft),u(t,x) = 0$
Case2	$V(t,y) = U_{\max } g(y)\sin (2\pi ft),u(t,x) = 0$	$V(t,y) = U_{\max } g(y)\cos (2\pi ft),u(t,x) = 0$

It can be identified from Fig. 4a–d that the blowing and suction process near the film hole seriously interferes with the internal flow, increasing the flow speed upstream. After the synthetic coolant is injected out of the orifice, the jet rolls up to form vortex structures. With the development of the flow field, when these vortices develop to a certain size, they will be merged with the flow field above the ground to form a certain protection for the bottom surface. Compared with the steady coolant, the distance between every two groups of vortices becomes farther when the synthetic coolant is incident, and the adhering force to the wall is smaller under various blowing ratio conditions. When the blowing ratio is small, most coolants are diffused into crossflow fluid in the vertical direction, instead of forming a protective gas film on the wall.

According to the comparison of the temperature contours at different blowing ratios in Fig. 4a–d, it can be inferred that the interactions between vortices are stronger at higher blowing ratios. Meanwhile, obvious mergers have occurred. In the process of these structures flowing downstream, the direction of these vortices gradually conform to the crossflow direction. And then they gradually dissipate into the ambient fluid due to the viscous effect, and the vorticity of the flow field is decreased.

According to the above simulation results, under the conditions of the fan-shaped hole incidence and *St* = 0.22, the film cooling effectiveness curves of different blowing ratios are displayed in Fig. 4e.

As indicated in Fig. 4e, the continuous vortex structures in the steady coolant flow field are changed by the synthetic coolant, resulting in the film cooling effectiveness curve unveiling obvious peaks and troughs. In the near field region, the entrainment is the strongest. As the coolant flows downstream, the engulfment becomes weaker or even disappeared, and the film cooling effectiveness curve is tending to flatten afterwards. However, different from the results of the steady coolant, the best film cooling performance of the synthetic coolant coincides with the blowing ratio of 2.5. This is because the higher the blowing ratios, the higher the gas momentum, and the more controllable the synthetic coolant can be. Compared with the steady coolant, the distance of synthetic coolant flowing along downstream gets longer under the same blowing ratio. For the reason that the entrainment of the synthetic coolant is stronger, the increasing rate of the temperature of the coolant core slows down, as the coolant extends into the downstream. As a result, the average adiabatic effectiveness in the downstream region is greater than that of the steady coolant ejected.

### Film cooling effectiveness of synthetic coolants incidence under different hole geometries

Similarly, the influence of hole geometry under the synthetic coolant incidence is discussed in the following, when the blowing ratio is fixed at 2.5 and *St* is taken as 0.22. The hole patterns are divided into square hole, round hole, fan-shaped hole, and two elliptical holes with respectively the aspect ratio of 0.618 and the other of 1.618. The simulation results are depicted in Fig. 5.

From Fig. 5a–e, it can be intuitively told that for synthetic coolant ejected, the cooling results of the fan-shaped hole and the two elliptical holes are greater in film coverage than the rest. Comparing the film cooling effects of two elliptical coolants, it is demonstrated that the vortex shedding at the leading edge of the low-aspect-ratio elliptical coolant (i.e. AR = 0.618) is more regular, and the interaction between the vortices and the vortex pairing phenomenon are more obvious. On the contrary, the vortex pairing does not seem to be observed for high-aspect-ratio elliptical coolants (i.e. AR = 1.618). Partly because there is a smaller curvature at the leading edge in the low-aspect-ratio elliptical coolant, which can move along streamwise at a relatively high speed. While the vortices in the flow field of high-aspect-ratio elliptical coolant are less likely to entangle and be paired.

According to the above results, under the conditions of the blowing ratio equaling to 2.5 and *St* = 0.22, the film cooling effectiveness of different hole patterns are expounded in detail in Fig. 5f.

Judging from the trend of the entire film cooling effectiveness curve in Fig. 5f, there is several sudden increases and decreases in fluid momentum during the downstream propagation. After these fluctuations, the curve flattens out.

However, different from the steady coolant ejected, according to the comparison chart of the film cooling effectiveness curve, it can be discovered that when the synthetic coolant is ejected from the elliptical hole with an aspect ratio of 0.618, it enjoys the largest velocity, and the directivity of the coolant is the strongest. The peaks and valleys change more drastically, and the average film cooling effectiveness is the highest. Apart from the larger momentum that the synthetic coolant possesses, there are also other reasons as mentioned above that different hole shapes correspond to their specific optimal blowing ratios. In contrast, when a round synthetic coolant is incident, the velocity of the coolant along downstream is the smallest. As a result, the coolant directivity is the weakest, and the film cooling effect is the worst.

### Film cooling effectiveness of the synthetic coolant incidence at different Strouhal numbers

Based on the investigation by Lalizel^26^ on the effect of Strouhal number excitation on film cooling efficiency, in this part, altering the non-stationarity the synthetic coolant is by changing the amplitude of the synthetic coolant, which can be accomplished by changing Strouhal number. The influence of the Strouhal number variation on the film cooling effectiveness is further analyzed right after. The conditions are set as the fan-shaped hole incidence, the blowing ratio is fixed at 2.5, and the Strouhal number is changed from 0.11 to 0.33. The temperature contours and vorticity magnitude contours are presented in Fig. 6a–c.

Comparing the two cases of *St* = 0.22 and *St* = 0.11, it can be conveyed from the temperature contours and vorticity magnitude contours that when the Strouhal number is small, the entrainment effect of the vortices on the surrounding fluid is enhanced, the acting region is large and the length of the vortex is long. The coolant penetrates deeply into the mainstream, resulting in the film cooling effectiveness deteriorated. When the Strouhal number increases to 0.33, the vortex intensity at the hole exit also is increased, the interaction between vortices becomes stronger, with hardly independent vortex structures visible, and the film cooling effectiveness decreases.

According to the above simulation results, under the conditions of the blowing ratio being 2.5, fan-shaped hole incidence, the comparison of different Strouhal numbers on the film cooling effectiveness is viewed in Fig. 6d.

It can be drawn from the research scope of this manuscript that when Strouhal number is 0.22, both the crossflow and coolant are regular flows. At this point, the interaction between the crossflow and the coolant is the strongest. Besides, this condition is the easiest to control among the three Strouhal numbers, so that it is admirable to take *St* = 0.22 to achieve the best film cooling performance.

### Effect of the sequence of dual-row incidence on film cooling effect

Based on the investigation of Izmodenova^27^ et al. on the dual-row incident cooling flow, this manuscript proposes two new cases, which have a same arrangement and different incident function. The arrangement of staggered double rows is used in two cases. In Case 1 of Table 1, the incident function between the left holes and the right holes is the same. While in Case 2, the incident function of the right row of holes is a cosine function, seen in Table 1 for details. That is, there is the same increase and decrease at Case1 for the two rows of holes, while different increase and decrease occurs in Case 2. And the film cooling effect of these two situations are separately conferred about and compared. The results are revealed in Fig. 7a–b.

The film cooling effectiveness curves corresponding to the two kinds of orders for the synthetic coolant under *St* = 0.22 are divulged in Fig. 7c.

By jointly analyzing the temperature contours and film cooling effectiveness curves, it is evident from the figures that when the stable film is formed, the cooling effect of the two in the entire fluid action zone is almost completely symmetrical, and the two film cooling curves are roughly in a complementary relationship. Due to the instability of the coolant and the complexity of the flow structures, at ‘5 < *x*/*D* < 7’, there are more peaks in the inserted dual-row incident coolants. If the film cooling effectiveness in the near field is given priority, simultaneous injection is more recommended. But in view of the far field cooling performance of staggered injection appeared a better state, it is possible to try to combine and alternately distribute these two kinds of injection modes, which can not only ensure the film cooling effectiveness in the near field, but can also improve the far field cooling.

## Conclusions

In this manuscript, the film cooling effect of synthetic coolant is numerically investigated, the corresponding film cooling effectiveness under the conditions of different blowing ratios, different hole geometries and different Strouhal numbers are analyzed, the film cooling characteristics of the two dual-row incident methods are also discussed and compared.

The main conclusions are summarized as follows: compared with the steady coolant ejected, the permeability of the synthetic coolant is notably enhanced, so is the gas entrainment, and the core temperature of the coolant is increased as the film expanded. When the Strouhal numbers are too small or too large, the vorticity at the exit becomes stronger, the vortex core length grows to be longer, and the film cooling effectiveness declines. At the same time, when the inserted dual-row coolants are incident, the trend of the film cooling effectiveness of the two kinds of dual-coolant injection sequences exhibits a complementary relationship. That is, the dual-row incidence of the same rule performs better in the near field region, in contrast, the other condition accords with better far field film cooling effect. It is worth noting that, for the both cases, the cooling performance of the staggered and aligned incidences are highly complementary to each other, and the combination of the two may further enhance the film cooling effectiveness of the near and far fields simultaneously.

The main conclusions being arrived at from the influence of different factors on the film cooling effectiveness when the synthetic coolant is ejected can be presented in Table 2.

**Table 2:** Comparison of the influence of different factors on the film cooling of the synthetic coolant.

	Streamwise film coverage	Spanwise film coverage	Film cooling effectiveness
**Hole shape**			
Round hole	D	D	D
Square hole	C−	C−	C−
Fan-shaped hole	B−	B	B−
Elliptical hole (AR = 0.618)	B+	B	B+
Elliptical hole (AR = 1.618)	B	B	B
**Strouhal number**			
*St* = 0.11	B−	B−	B−
*St* = 0.22	A	A	A
*St* = 0.33	C	C	C
**Blowing ratio**			
M = 0.5	C	B	B+
M = 1	B	C−	C
M = 2	B	B	B
M = 2.5	A	A	A
